# Clinical profile, management strategy, and outcomes of patients with prosthetic valve thrombosis

**DOI:** 10.21542/gcsp.2024.44

**Published:** 2024-11-01

**Authors:** Waqar Khan, Arsalan Younus, Muhammad Imran Ansari, Jehangir Ali Shah, Mariam Naz, Raheela Khawaja, Aamir Khowaja, Taimur Asif Ali, Munawar Khursheed, Tahir Saghir

**Affiliations:** National Institute of Cardiovascular Diseases (NICVD), Karachi, Pakistan

## Abstract

**Background:** Prosthetic valve thrombosis (PVT) is a severe complication following prosthetic heart valve replacement, particularly in inadequately anticoagulated patients. Primary treatment options include intensive anticoagulation therapy, thrombolytic treatment (TT), and emergency surgery. This study aims to evaluate the clinical profile, management strategies, and short-term outcomes of patients with PVT.

**Methodology:** Consecutive patients with PVT presenting to the emergency department of a tertiary care cardiac center were included in this study. Responses to treatment, hospital outcomes, and 30-day outcomes post-treatment were observed.

**Results:** A total of 75 patients were analyzed, with a male predominance (50.7%) and a mean age of 39.5 ± 12.3 years. Bi-leaflet prosthetic valves were most common (96.0%), 54 (72.0%) had prosthetic mitral valve and 10 (13.3%) had prosthetic both mitral and aortic valves. Atrial fibrillation was present in 25.3% of cases. Treatment predominantly involved streptokinase (74.7%), followed by heparin (37.3%) and VKA (9.3%). Clinical success was achieved in 84.0% of cases, while 12.0% experienced clinical failure, including severe complications such as irreversible neurologic damage (1.3%) and bleeding (2.8%). The 30-day mortality rate was 12.0%, with recurring PVT and bleeding/embolic complications each in 1.5% of cases.

**Conclusion:** Treatment of PVT with streptokinase, heparin, and VKA demonstrates efficacy, with a substantial proportion of patients achieving complete clinical success. However, the study highlights concerning outcomes, including clinical failure and severe complications. These findings underscore the importance of carefully balancing thrombolytic and anticoagulant therapies to mitigate potential adverse events.

## Introduction

The prevalence of valvular heart disease (VHD) varies globally, with rheumatic heart disease (RHD) affecting approximately 41 million individuals worldwide^1,2^. Calcific aortic valve disease (CAVD) has also become increasingly common, leading to rising incidence, prevalence, and mortality rates over the past three decades^3,4^. While non-rheumatic VHD has been steadily increasing, the occurrence of RHD has been on the decline^1,5^. Contributing factors include high systolic blood pressure, aging populations, and lifestyle choices such as smoking and alcohol consumption^3,6^.

For patients with severe native valvular heart disease, surgical replacement with mechanical or biological valves, as well as percutaneous valve implantation, are established gold standard therapies^7,8^. However, prosthetic heart valve replacement is not without its complications, both during and after the procedure. Despite the long-term durability of mechanical valves, valve malfunction remains a concern, with mechanical prosthetic valve thrombosis (PVT) being a particularly serious complication that can lead to rapid deterioration and compromise patient outcomes^9^.

PVT is an uncommon yet potentially life-threatening condition that can occur in any part of the heart valve prosthesis^10^. Although the exact incidence of valve thrombosis is uncertain, estimates suggest it ranges from 0.5% to 6% per patient per year in the aortic and mitral positions, and up to 20% in the tricuspid position^11^. Factors influencing PVT development include inadequate anticoagulant treatment, valve thrombogenicity, and hemodynamics of transprosthetic blood flow^12^. Despite advancements in surgical techniques, mechanical valves’ hemodynamic and physical properties still pose a risk for thrombus formation^13^.

While emergency surgery has been the traditional treatment for PVT, intravenous thrombolytic therapy has emerged as a viable alternative, showing excellent success rates and acceptable risks^14^. Current guidelines recommend either slow-infusion low-dose thrombolytic therapy (TT) or emergency surgery for PVT treatment^15^. In settings like Pakistan, where regular INR monitoring may be challenging due to geographic and financial constraints, thrombolytic therapy remains the primary treatment due to financial considerations. Therefore, this study aims to evaluate the clinical characteristics, management strategies, and short-term outcomes of patients presenting with prosthetic valve thrombosis at a tertiary care cardiac center’s emergency department.

## Methodology

**Study design:** We conducted this prospective observational study at the emergency department of the largest tertiary care cardiac center in Karachi, Pakistan, the National Institute of Cardiovascular Diseases (NICVD). Our aim was to assess the clinical profile, opted management strategy, and in-hospital and 30-day outcomes of patients presenting with PVT. Data collection was carried out between November 22, 2021 and November 21, 2022.

**Ethics:** The study was conducted in accordance with the guidelines provided by the Declaration of Helsinki. Verbal consent for participation in the study was obtained from all participants prior to their inclusion. The study proposal was approved by the institution review board of the NICVD (ERC-119/2021).

**Study population:** The study population consisted of a consecutive sample of adult patients (≥ 18 years) of either gender who presented with PVT. Patients who declined consent for participation or those who left against medical advice (LAMA) were excluded from the analysis.

**Data collection:** Data for the study were collected using a predefined structured proforma. Collected data included demographic information such as age, gender, NYHA (New York Heart Association) class, and disease-related factors such as time since valve replacement, type of prosthetic valve, position of PVT, prosthesis size, atrial fibrillation, echocardiography parameters such as thrombus size, LV (left ventricular) dysfunction, RV (right ventricular) dysfunction, TR, trans-valvular gradient (mean/peak), and anticoagulation status. Additionally, data regarding the opted management strategy such as streptokinase, heparin, VKA, emergency surgery, and in-hospital outcomes of the patients such as hemodynamic response, clinical success, and incidence of any major embolic complication during hospital stay and post-discharge 30-day follow-up were also recorded.

Patients without contraindications for streptokinase received: loading dose of 250,000 International Units (IU) over 1 h and maintenance dose of 100,000 IU/hour *via* continuous infusion. For patients with contraindications to thrombolytic therapy, anticoagulation was initiated with heprin infusion with Targeted Partial Thromboplastin Time (PTT) of 1.5-2 times the upper normal range. PTT was repeated every 6 h.

At 24 h, a detailed echocardiogram, Hemodyanamic and clinical response was noted. Before discharge patient were bridged to warfarin and targeted INR achieved. Weekly INR monitoring was scheduled.

**Variables and definitions:** The hemodynamic response was categorized as follows: complete response - normalization of the trans-valvular mean and peak gradients (>75% reduction) on Doppler echocardiography and restoration of normal leaflet(s) motion on cine-fluoroscopy; partial response – 50–75% reduction in trans-valvular gradients with restricted movement of prosthetic valve leaflet(s) on cine-fluoroscopy, even if gradients decreased by more than 75%; hemodynamic failure – less than 50% reduction in trans-valvular gradients. Clinical success was categorized as follows: complete clinical success - complete hemodynamic response in the absence of any major complication; partial success - either complete or partial hemodynamic response and occurrence of any major hemorrhagic/embolic complication; clinical failure - either hemodynamic failure or occurrence of a complication resulting in death irrespective of the hemodynamic response. A major embolic complication was defined as one resulting in irreversible neurologic damage or myocardial infarction or one needing limb-salvage surgery.

**Sample size:** A total of 75 patients presenting to the emergency department with PVT during a 12-month study period were included in this study. The collected sample was deemed sufficient due to the exploratory nature of the analysis; however, no formal calculation of sample size was carried out.

**Data Analysis:** The collected data were summarized in accordance with the study objective. Appropriate summary measures such as mean ± standard deviation (SD) or median [interquartile range (IQR)] were computed for age, INR level, length of stay, and echocardiographic parameters. The distribution of various clinical and demographic variables along with outcome variables was expressed as percentages (%). Pre- and post-echocardiographic parameters were compared with paired sample *t*-test or Chi-square test, with the significance level set at *p* ≤ 0.05. The univariable and stepwise forward conditional multivariable binary logistic regression analysis was performed for clinical failure or partial response and odds ratio (OR) along with 95% confidence interval (CI) were computed.

## Results

A total of 75 patients were included in this analysis; 38 (50.7%) were male, and the mean age was 39.5 ± 12.3 years. The type of prosthetic valve was bi-leaflet in 72 (96.0%) patients, with 54 (72.0%) having a prosthetic mitral valve and 10 (13.3%) having prosthetic valves in both the mitral and aortic positions. Atrial fibrillation was prevalent in 25.3% (19 patients), and 6 (8.1%) patients had a history of CVA/stroke. Among the patients, 56 (74.7%) were treated with streptokinase, 28 (37.3%) received heparin, and VKA was administered in 7 (9.3%) patients (Table 1).

**Table 1:** Distribution of clinical characteristics, prosthetic valve details, and management strategy among patients with prosthetic valve thrombosis.

	**Summary**
**Total (N)**	**75**
**Gender**	
Male	38 (50.7%)
Female	37 (49.3%)
**NYHA Class**	
I	2 (2.7%)
II	40 (53.3%)
III	24 (32.0%)
IV	9 (12.0%)
**Age (years)**	39.5 ± 12.3
**Type of prosthetic valve**	
Ball and cage	3 (4.0%)
Bi-leaflet	72 (96.0%)
**Prosthetic valve**	
Mitral	54 (72.0%)
Aortic	11 (14.7%)
Both	10 (13.3%)
**Atrial fibrillation**	19 (25.3%)
**History of stroke/CVA**	6 (8.1%)
**Thrombus before treatment**	3 (4.0%)
**Management strategy PVT**	
Streptokinase	56 (74.7%)
Heparin	28 (37.3%)
Vitamin K antagonists (VKA)	7 (9.3%)

**Notes.**

CVA cerebral vascular accident PVT prosthetic valve thrombosis

The mean INR level increased from 1.8 ± 1.2 to 2.4 ± 0.7 ( *p* < 0.001) after treatment. Both the mean and peak trans-valvular gradients across the mitral valve decreased significantly from 21.0 ± 12.4 to 10.8 ± 10.7 (*p* < 0.001) and 30.7 ± 15.7 to 16.0 ± 13.1 (*p* < 0.001), respectively. Similarly, both the mean and peak trans-valvular gradients across the aortic valve decreased significantly from 39.8 ± 24.9 to 17.2 ± 9.0 (*p* < 0.001) and 59.9 ±39.7 to 24.8 ± 13.2 (*p* < 0.001) (Table 2).

**Table 2:** Comparison of INR level and echocardiographic findings before and after treatment of patients with prosthetic valve thrombosis.

	**Before Treatment**	**After Treatment**	**P-value**
**INR**	1.8 ± 1.2	2.4 ± 0.7	<0.001
**Left ventricular dysfunction**			
None	53 (70.7%)	53 (70.7%)	0.971
Mild	3 (4.0%)	2 (2.7%)	
Moderate	13 (17.3%)	14 (18.7%)	
Severe	6 (8.0%)	6 (8.0%)	
**Right ventricular dysfunction**			
None	60 (80.0%)	59 (78.7%)	0.997
Mild	2 (2.7%)	2 (2.7%)	
Moderate	10 (13.3%)	11 (14.7%)	
Severe	3 (4.0%)	3 (4.0%)	
**Tricuspid regurgitation**			
None	52 (69.3%)	51 (68.0%)	0.995
Mild	8 (10.7%)	8 (10.7%)	
Moderate	7 (9.3%)	8 (10.7%)	
Severe	8 (10.7%)	8 (10.7%)	
**Mitral valve: trans-valvular mean and peak gradients**			
Mean (mmHg)	21.0 ± 12.4	10.8 ± 10.7	<0.001
Peak (mmHg)	30.7 ± 15.7	16.0 ± 13.1	<0.001
**Aortic valve: trans-valvular mean and peak gradients**			
Mean (mmHg)	39.8 ± 24.9	17.2 ± 9.0	<0.001
Peak (mmHg)	59.9 ± 39.7	24.8 ± 13.2	<0.001

**Notes.**

INR international normalised ratio

Complete hemodynamic response was noted in 63 (84.0%) patients, while 9 (12.0%) patients experienced hemodynamic failure. Similarly, 63 (84.0%) patients achieved complete clinical success, whereas 9 (12.0%) patients encountered clinical failure, among whom one patient (1.3%) suffered irreversible neurologic damage, and 2 (2.8%) patients experienced bleeding complications. The 30-day mortality rate was 12.0% (9/75), with one patient experiencing PVT recurrence, one patient encountering PVT (1.5%), and one patient experiencing bleeding/embolic complications (1.5%) (Table 3).

**Table 3:** Distribution of post treatment in-hospital and 30-day outcomes of patients with prosthetic valve thrombosis.

	**Summary**
**Total (N)**	**75**
**Hemodynamic response**	
Complete response	63 (84.0%)
Partial response	3 (4.0%)
Hemodynamic failure	9 (12.0%)
**Clinical success**	
Complete success	63 (84.0%)
Partial success	3 (4.0%)
Clinical failure	9 (12.0%)
**Major embolic complication**	
Irreversible neurologic damage	1 (1.3%)
**Bleeding complication**	2 (2.8%)
Bleeding gums	1 (1.3%)
Hematuria	1 (1.3%)
**Length of Stay (days); Median (IQR)**	4.0 (3.0–6.0)
**30-day outcomes**	
All-cause mortality	9 (12.0%)
Re-current hospitalization	1 (1.5%)
Prosthetic valve thrombosis	1 (1.5%)
**Functional class**	
I	54 (81.8%)
II	12 (18.2%)
Bleeding/embolic complications	1 (1.5%)

The multivariable analysis revealed NYHA class III and IV were independently associated with higher risk of clinical failure or partial response with adjusted ORs of 21.2 [1.57–285.99] and 543.62 [18.05–16369.15], respectively. While, administration of Streptokinase was indendently associated with a lower risk of clinical failure or partial response with adjusted OR of 0.03 [0.003–0.43] (Table 4).

**Table 4:** Univariable and multivariable binary logistic regression analysis for clinical failure or partial response.

** **	**Univariable**		**Multivariable (Stepwise: Forward Conditional)**	
	**OR [95% CI]**	**P-value**	**OR [95% CI]**	**P-value**
Male	2.2 [0.6–8.06]	0.234	–	–
**NYHA class**				
I/II	Reference	–	Reference	–
III	10.79 [1.18–98.83]	0.035	21.2 [1.57–285.99]	0.021
IV	82.0 [7.29–922.06]	<0.001	543.62 [18.05–16369.15]	<0.001
Age (years)	1.00 [0.95–1.05]	0.950	–	–
Atrial fibrillation	1.60 [0.42–6.07]	0.489	–	–
History of stroke/CVA	1.05 [0.11–9.92]	0.963	–	–
Thrombus before treatment	12.4 [1.03–149.81]	0.048	19.93 [0.74–534.78]	0.075
Streptokinase given	0.40 [0.11–1.46]	0.165	0.03 [0.003–0.43]	0.009
Heparin given	1.86 [0.54–6.47]	0.327	–	–
Baseline INR level	0.83 [0.41–1.69]	0.612	–	–
Left ventricular dysfunction	1.93 [0.54–6.92]	0.311	–	–
Right ventricular dysfunction	1.42 [0.33–6.04]	0.638	–	–
Tricuspid regurgitation	0.72 [0.18–2.94]	0.643	–	–

**Notes.**

OR odds ratio CI confidence interval CVA cerebral vascular accident

## Discussion

The study examined the clinical characteristics, management approaches, and short-term outcomes of patients presenting with PVT following prosthetic heart valve replacement. Among the 75 patients analyzed, predominantly male with a mean age of 39.5 years, bi-leaflet prosthetic valves were most common, with the mitral valve being predominantly affected. A significant proportion of patients exhibited atrial fibrillation. Treatment predominantly comprised streptokinase, with notable success rates. A substantial proportion of patients (84.0%) achieved complete clinical success, underscoring the effectiveness of thrombolytic therapy. However, the study also revealed concerning outcomes, including clinical failure in 12.0% of patients, with one case resulting in irreversible neurologic damage. Additionally, bleeding complications were observed in a small percentage of patients. The success rate of 84% in our study aligns with the success rate reported by various studies. The reported success rate of thrombolytic therapy ranges from 65% to 92.4%^16–23^.

Moreover, the 30-day mortality rate of 12.0% underscores the gravity of PVT as a life-threatening complication, emphasizing the need for prompt and effective management strategies to improve patient outcomes. Furthermore, the recurrence of PVT and bleeding/embolic complications within the short follow-up period emphasize the necessity of long-term monitoring and comprehensive management strategies to mitigate the risk of recurrent events. These findings highlight the importance of balancing the benefits of thrombolytic and anticoagulant therapies with the potential risks of adverse events, particularly in patients with PVT. The recurrence of PVT after thrombolytic therapy remains an issue, with incidence varying from 16.2% to 31%^20,24,25^. In our study, we observed recurrence of PVT in 1 patient during the 30-day follow-up.

Similar to our study, Manandhar R et al.^26^ conducted a retrospective study of 45 cases presented to a cardiac center in Nepal over a period of 2 years. Atrial fibrillation was noted in 46.7%, and PVT was mostly at the mitral position (87%). A majority, 86.9%, were managed with thrombolysis, with streptokinase being the main treatment modality (86.9%), and in-hospital mortality was reported to be 13.3%, with no major bleeding or new stroke events. Hirachan A et al.^16^ reported suboptimal anticoagulation (INR < 1.5) in 69.6% of the case series of 23 patients with PVT. Thrombolysis, primarily with streptokinase, was administered to 86.9% of patients. Mitral valve thrombosis was most common (73.9%). In-hospital mortality occurred in 21.7% of cases, with no major bleeding events or new strokes noted.

Sharma V et al.^17^ concluded that thrombolysis is a reasonable option for PVT management, especially in cases of warfarin poor compliance and subtherapeutic INR. The study reported the efficacy of both tPA or SK with a success rate of 86.66% with tPA, while partial success and failure were observed in 17.77% and 6.66% respectively. For patients treated with streptokinase (*n* = 27), complete success was reported in 85.19%, with partial success and failure in 11.11% and 3.7% respectively. In another study from India by Kiran GR et al.^18^, the efficacy of streptokinase and tenecteplase for patients with PVT was compared. The complete hemodynamic response and complete clinical success were observed in 81% and 84.5% of the cases with 8.3% bleeding events and 4.7% embolic manifestations. However, tenecteplase was found to be associated with a lower complication rate. Continuing on the same premise, Kathirvel D et al.^21^ evaluated the efficacy and safety of tenecteplase and streptokinase for the treatment of PVT. Slow infusion of tenecteplase is equally efficacious but more effective than streptokinase in managing PVT with a complete success rate of 77.5% *vs.* 75%, respectively. However, minor bleeding (16.7% *vs.* 0%) was more common in tenecteplase compared to streptokinase, respectively. Hence, thrombolytic therapy should be considered as the first-line therapy when immediate surgical options are not feasible.

A study by Mahindru S et al.^19^ evaluated the midterm follow-up of patients with PVT (stuck valve), focusing on outcomes after thrombolysis. Thrombolysis was successful in 92.4% with a mortality rate of 7.57%. However, the 5-year mortality rate was found to be 22.95%. Milne O et al.^20^ conducted a retrospective case series of 21 patients with 32 episodes during a 17-year period at a cardiac center in the Northern Territory of Australia. The majority of patients presented with severe symptoms (NYHA class III and IV) and subtherapeutic anticoagulation (88%). Most valves were mechanical, with an average time from implantation to initial PVT of 5.1 years. Thrombolytic therapy was the main treatment approach (82% of episodes), achieving complete success in 65% and partial success in 19%. However, four patients did not respond to thrombolytic therapy, resulting in mortality or urgent transfer to a facility with cardiothoracic surgery capabilities. Overall mortality for the cohort was 24%, with thrombolytic therapy associated with major bleeding episodes in 16% of cases.

The low-dose and slow infusion thrombolytic therapy is a safe and effective management strategy in elderly patients with PVT. In a study by Gündüz S et al.^22^, this strategy has a cumulative success rate of 85.2%, with adverse events in 22.2% of patients. Higher thrombus burden and New York Heart Association class were predictive of adverse events, with the thrombus area being the only independent predictor. A study by Raman K et al.^27^ argued the effect of reoperation *vs.* thrombolysis on the long-term outcomes of patients with PVT. The reoperation was found to be advantageous over thrombolysis, with a significantly lower rate of embolism, bleeding events, re-intervention, as well as mortality at the end of 10-years follow-up.

The main factors influencing the development of PVT are inappropriate treatment by anticoagulants, thrombogenicity of the valve, and hemodynamics of the transprosthetic blood flow. A study by Bezanjani FN et al.^28^ reported several factors significantly associated with thrombosis included inadequate anticoagulation (INR < 2.5), a history of infection, prothrombin time check interval, atrial fibrillation rhythm, and plasma fibrinogen level.

It is important to acknowledge certain limitations of the study. It was conducted at a single tertiary care cardiac center and included a relatively small sample size of 75 patients. The study primarily focused on short-term outcomes up to 30 days post-treatment, which may not capture long-term complications or outcomes, limiting the comprehensive evaluation of treatment efficacy and safety over time. Finally, the study lacks a control or comparison group, making it challenging to assess the relative effectiveness of different treatment modalities or to compare outcomes with alternative management strategies. Hence we recommend prospective studies with larger sample size, longer follow-up period, and multi-center design to overcome the shortcoming of this study.

## Conclusion

In conclusion, this study provides valuable insights into the clinical characteristics, management approaches, and short-term outcomes of patients presenting with PVT. While the majority of patients achieved successful outcomes with appropriate interventions, the study also highlights the challenges and potential complications associated with PVT management. Further research is warranted to elucidate optimal treatment strategies and long-term outcomes in this patient population.

## References

[1] Yu J, Wang Z, Bao Q, Lei S, You Y, Yin Z, Xie X (2022). Global burden of calcific aortic valve disease and attributable risk factors from 1990 to 2019. Front Cardiovasc Med.

[2] Aluru JS, Barsouk A, Saginala K, Rawla P, Barsouk A (2022). Valvular Heart Disease Epidemiology. Med Sci (Basel).

[3] Zheng X, Guan Q, Lin X (2023). Changing trends of the disease burden of non-rheumatic valvular heart disease in China from 1990 to 2019 and its predictions: Findings from global burden of disease study. Front Cardiovasc Med.

[4] Yi B, Zeng W, Lv L, Hua P (2021). Changing epidemiology of calcific aortic valve disease: 30-year trends of incidence, prevalence, and deaths across 204 countries and territories. Aging (Albany NY).

[5] Santangelo G, Bursi F, Faggiano A, Moscardelli S, Simeoli PS, Guazzi M, Lorusso R, Carugo S, Faggiano P (2023). The Global Burden of Valvular Heart Disease: From Clinical Epidemiology to Management. J Clin Med.

[6] Chen J, Li W, Xiang M (2020). Burden of valvular heart disease, 1990-2017: Results from the Global Burden of Disease Study 2017. J Glob Health.

[7] Writing Committee Members, Otto CM, Nishimura RA, Bonow RO, Carabello BA, Erwin3rd JP, Gentile F, Jneid H, Krieger EV, Mack M, McLeod C, O’Gara PT, Rigolin VH, Sundt3rd TM, Thompson A, Toly C (2021). 2020 ACC/AHA Guideline for the Management of Patients with Valvular Heart Disease: Executive Summary: A Report of the American College of Cardiology/American Heart Association Joint Committee on Clinical Practice Guidelines. J Am Coll Cardiol.

[8] Falk V, Baumgartner H, Bax JJ, De Bonis M, Hamm C, Holm PJ, Iung B, Lancellotti P, Lansac E, Muñoz DR, Rosenhek R, Sjögren J, Tornos Mas P, Vahanian A, Walther T, Wendler O, Windecker S, Zamorano JL, ESC Scientific Document Group (2017). 2017 ESC/EACTS Guidelines for the management of valvular heart disease. Eur J Cardiothorac Surg.

[9] Hassouna A, El-Ghanam M, Moftah H, Samir K, Refaat K (2020). Index of deterioration of patients with mechanical prosthetic heart valve thrombosis. Cardiothorac Surg.

[10] Capodanno D, Petronio AS, Prendergast B, Eltchaninoff H, Vahanian A, Modine T, Lancellotti P, Sondergaard L, Ludman PF, Tamburino C, Piazza N, Hancock J, Mehilli J, Byrne RA, Baumbach A, Kappetein AP, Windecker S, Bax J, Haude M (2017). Standardized definitions of structural deterioration and valve failure in assessing long-term durability of transcatheter and surgical aortic bioprosthetic valves: a consensus statement from the European Association of Percutaneous Cardiovascular Interventions (EAPCI) endorsed by the European Society of Cardiology (ESC) and the European Association for Cardio-Thoracic Surgery (EACTS). Eur Heart J.

[11] Yaminisharif A, Alemzadeh-Ansari MJ, Ahmadi SH (2012). Prosthetic tricuspid valve thrombosis: three case reports and literature review. J Tehran Heart Cent.

[12] Sr K, Bharath G, Jain S, Moorthy N, Manjunath SC, Christopher R (2019). Prosthetic valve thrombosis - association of genetic polymorphisms of VKORC1, CYP2C9 and CYP4F2 genes. Medicine (Baltimore).

[13] Lim WY, Lloyd G, Bhattacharyya S (2017). Mechanical and surgical bioprosthetic valve thrombosis. Heart.

[14] Cáceres-Lóriga FM, Morais H (2015). Thrombotic obstruction in left-side prosthetic valves: Role of thrombolytic therapy. Indian Heart J.

[15] Nishimura RA, Otto CM, Bonow RO, Carabello BA, Erwin3rd JP, Fleisher LA, Jneid H, Mack MJ, McLeod CJ, O’Gara PT, Rigolin VH, Sundt3rd TM, Thompson A (2017). 2017 AHA/ACC Focused Update of the 2014 AHA/ACC Guideline for the Management of Patients With Valvular Heart Disease: A Report of the American College of Cardiology/American Heart Association Task Force on Clinical Practice Guidelines. Circulation.

[16] Hirachan A, Roka M, Prajapati D, Adhikari CM, Bishal KC, Khadka MB, Maskey A, Sharma D (2017). Prosthetic valve thrombosis in a tertiary cardiac centre. Nepalese Heart Journal.

[17] Sharma V, Arora BY, Gupta LC, Poonia A, Raina S, Yadav US, Sharma R, Dwivedi S (2022). The efficacy and safety of thrombolytic agents for patients with prosthetic valve thrombosis. J Prac Cardiovasc Sci.

[18] Kiran GR, Chandrasekhar P, Mohammad Ali S (2021). Clinical outcomes of patients with mitral prosthetic valve obstructive thrombosis treated with streptokinase or tenecteplase. Ind Heart J.

[19] Mahindru S, Pande S, Malhotra P, Thukral A, Kotwal AS, Gupta RP, Garg N, Kapoor A, Agarwal SK (2021). Mechanical prosthetic valve thrombosis in current era: 5-year follow-up. Indian J Thorac Cardiovasc Surg.

[20] Milne O, Barthwal R, Agahari I, Ilton M, Kangaharan N (2020). Management and Outcomes of Prosthetic Valve Thrombosis. An Australian Case Series From the Northern Territory. Heart Lung Circ.

[21] Kathirvel D, Justin Paul G, Prathap Kumar G, Palanisamy G, Gnanavelu G, Ravishankar G, Swaminathan N, Venkatesan S (2018). Tenecteplase versus streptokinase thrombolytic therapy in patients with mitral prosthetic valve thrombosis. Indian Heart J.

[22] Gündüz S, Özkan M, Yesin M, Kalçık M, Gürsoy MO, Karakoyun S, Astarcıoğlu MA, Aykan AÇ, Gökdeniz T, Biteker M, Duran NE, Yıldız M (2017). Prolonged Infusions of Low-Dose Thrombolytics in Elderly Patients with Prosthetic Heart Valve Thrombosis. Clin Appl Thromb Hemost.

[23] Roudaut R, Lafitte S, Roudaut MF, Courtault C, Perron JM, Jaïs C, Pillois X, Coste P, De Maria A (2003). Fibrinolysis of mechanical prosthetic valve thrombosis: a single-center study of 127 cases. J Am Coll Cardiol.

[24] Keuleers S, Herijgers P, Herregods MC, Budts W, Dubois C, Meuris B, Verhamme P, Flameng W, Van de Werf F, Adriaenssens T (2011). Comparison of thrombolysis versus surgery as a first line therapy for prosthetic heart valve thrombosis. Am J Cardiol.

[25] Cáceres-Lóriga FM, Pérez-López H, Morlans-Hernández K, Facundo-Sánchez H, Santos-Gracia J, Valiente-Mustelier J, Rodiles-Aldana F, Marrero-Mirayaga MA, Betancourt BY, López-Saura P (2006). Thrombolysis as first choice therapy in prosthetic heart valve thrombosis. A study of 68 patients. J Thromb Thrombolysis.

[26] Manandhar R, Prajapati D, Tamrakar R, Bogati A, Roka M, Sherpa K, Shrestha S, Bhandari S,  Chaudhary T, Yadav S, Adhikari CM (2022). Clinical profile and management of prosthetic valve thrombosis in Tertiary cardiac Centre of Nepal, a prospective study. Nepalese Heart Journal.

[27] Raman K, Mohanraj A, Palanisamy V, Ganesh J, Rawal S, Kurian VM, Sethuratnam R (2019). Thrombolysis and surgery for mitral prosthetic valve thrombosis: 11-year outcomes. Asian Cardiovasc Thorac Ann.

[28] Noohi Bezanjani F, Gohari S, Bassiri HA, Ahangar H, Reshadmanesh T (2020). Risk Factors Associated with Heart Valve Thrombosis in Patients with Prosthetic Heart Valve Dysfunction. Arch Iran Med.

